# Biomass fuel usage for cooking and frailty among older adults in China: a population-based cohort study

**DOI:** 10.3389/fpubh.2023.1122243

**Published:** 2023-04-12

**Authors:** Quhong Song, Miao Dai, Taiping Lin, Yanli Zhao, Xuchao Peng, Rui Liang, Qiaoli Su, Jirong Yue

**Affiliations:** ^1^Department of Geriatrics and National Clinical Research Center for Geriatrics, West China Hospital, Sichuan University, Chengdu, China; ^2^Department of Geriatrics, Jiujiang First People's Hospital, Jiujiang, China; ^3^Department of General Practice, West China Hospital, Sichuan University, Chengdu, China

**Keywords:** biomass fuels, household air pollution, frailty index, FRAIL scale, older people, cohort

## Abstract

**Background:**

Although outdoor air pollution is reported to have a negative effect on frailty, evidence involving household air pollution is sparse.

**Methods:**

A cohort study on older participants aged ≥65 years from the Chinese Longitudinal Healthy Longevity Survey was conducted between 2011/2012 and 2014. Household cooking fuel types were determined by self-reported questionaries, and were dichotomized into clean or biomass fuels. The frailty status was evaluated *via* a 46-item frailty index (FI) and the FRAIL scale, respectively. Frailty was identified if FI >0.21 or FRAIL score ≥3. Cox proportional hazards models were employed to examine the relationship between cooking fuels and incident frailty. And the effects of swapping cooking fuels on frailty risk were also explored.

**Results:**

Among 4,643 participants (mean age at baseline 80.9 ± 9.6 years, 53.7% male) totaling 11,340 person-years, 923 (19.9%) incident frailty was identified using FI. Compared to clean fuels, cooking with biomass fuels was intricately linked to a 23% rise in frailty risk (hazard ratio [HR] 1.23, 95% confidence interval [CI] 1.06–1.43). A similar association was detected between biomass cooking fuels and frailty measured by the FRAIL scale (HR 1.24, 95% CI 1.04–1.50). Sensitive analyses supported the independent relationship between biomass fuels and frailty. Stratified analyses revealed that the frailty risk was higher among town residents (HR 1.44, 95% CI 1.13–1.84) and participants not exercising regularly (HR 1.35, 95% CI 1.11–1.64). In comparison with persistent biomass fuels usage, switching to clean fuels had a trend to reduce the frailty risk, and the opposite effect was observed when swapping from clean to biomass fuels.

**Conclusion:**

Cooking with biomass fuels was associated with an increased frailty risk in older adults, especially amongst those living in town and those lacking regular exercise. More studies are needed to confirm our findings and to evaluate the potential benefits of reducing indoor biomass fuel usage.

## Introduction

Frailty is an age-dependent syndrome that diminishes functioning across multiple physiological systems while increases responsiveness to various stressors (1). It is intricately associated with numerous undesirable outcomes, including mortality (2), disability (3), falls (4), fractures (5), low quality of life (6), and increased health-care expenditure and utilization (7). With a rising aging population and a longer life expectancy, older people suffering from frailty are dramatically increasing. Frailty is emerging as a global health burden, posing great challenges to health, aging and healthcare system, particularly, in those low- and middle-income locations (1).

Being a populous country, China has a rapidly growing older population (8). According to the data published by the National Bureau of Statistics of China, in the year 2020, China had 264 million people aged 60 years or older, accounting for 18.7% of the total 1.41 billion people (9). And this population is estimated to be 28% by the year 2040, given the enhanced survival durations and vastly reduced fertility rates (8). The biggest challenge of population aging is frailty (10). A previous research, based on the China Health and Retirement Longitudinal Study (CHARLS), has revealed that frailty is more common at advanced ages, and there were 51.2% prefrail and 7.0% frail adults among those aged ≥60 years in 2011 (11), which greatly burdened the long-term medical care in China. A comprehensive insight into frailty risk factors may provide opportunities to prevent and manage frailty, especially when these risk factors are manageable.

Multiple sociodemographic, lifestyle, clinical, and biological conditions (e.g., low education, physical inactivity, multimorbidity) are correlated with frailty (1, 12). In recent epidemiologic studies, environmental factors like air pollution were demonstrated to enhance frailty risk among older adults (13–17). Nonetheless, current publications mostly focused on outdoor air pollution (14–17). Till date, only one research explored the association between household air pollution (HAP) and frailty (13). Although HAP from solid fuels has declined markedly over the last few years, 36% of the Chinese population still relies on biomass or coal fuels for cooking (18), which, unfortunately, accounts for ~1 million premature deaths in China each year (19). Given the rapidly aging population and the common usage of biomass fuels, assessing the influence of HAP from biomass fuels on frailty is both urgent and necessary.

Herein, we employed the Chinese Longitudinal Healthy Longevity Survey (CLHLS), a longitudinal survey designed for an exclusively Chinese population, to investigate the association between HAP from biomass cooking fuels and the risk of frailty. And we also assessed whether altering cooking fuel types has an effect on frailty risk.

## Methods

### Study population

This study was based on CLHLS, an ongoing, prospective, population-based cohort involving 23 of the 31 provinces in China. CLHLS was first initiated in 1998, and held subsequent follow-up interviews about every 2–3 years. Details of the CLHLS survey have been described in the previous publication (20). Here, we utilized data from the 2011/2012 wave of CLHLS, which included the question “Which fuels are normally used for cooking in your home?”, and the follow-up survey was performed in 2014.

**Figure 1:**
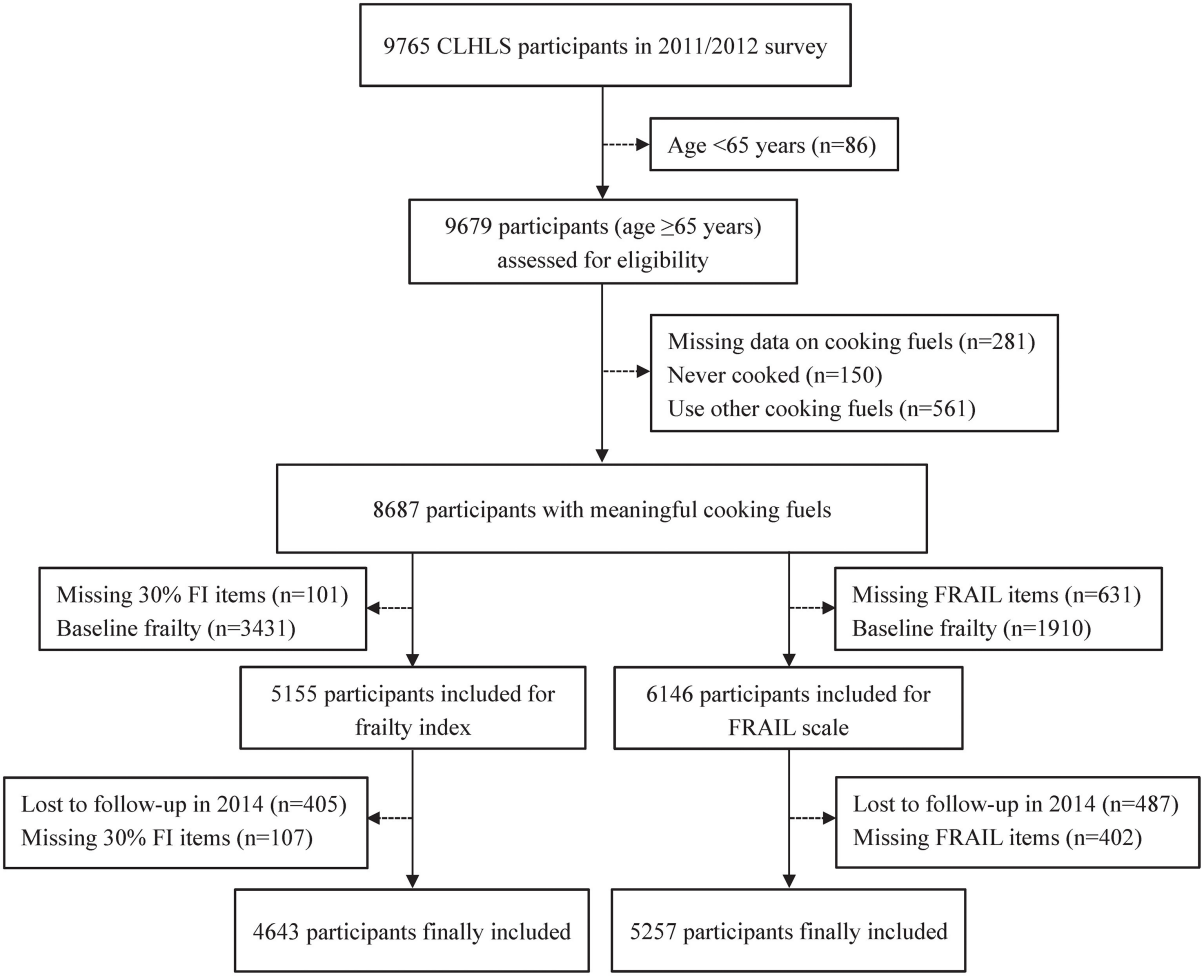
Flow chart of participants' selection. FI, frailty index.

The inclusion criteria were older adults aged ≥65 years. Participants were eliminated if they had any of the following conditions: (1) missing data on cooking fuels, (2) never cooking, or using other cooking fuels like fuel oil, kerosene, coal or coke, (3) missing key variables to determine frailty status, (4) baseline frailty, or (5) lost to follow-up. The inclusion and exclusion processes were detailed in Figure 1.

The study received ethical approval from Peking University, Beijing, China (IRB00001052-13074), and closely followed the standards of the Declaration of Helsinki. Documented informed consent was received from subjects or their legal representatives prior to the initiation of the study.

### Household cooking fuel types

The primary fuel types used in cooking was determined by self-reported questionnaires that were answered at baseline and during follow-up, and were categorized into clean or biomass fuels. Biomass cooking fuels included straw, firewood, and charcoal, while clean fuels comprised gas, solar energy, and electricity. Using wave-specific data, the shifting of cooking fuels during follow-up was also evaluated, and participants were then divided into four categories: persistent clean fuels, clean to biomass fuels switch, persistent biomass fuels, and biomass to clean fuels switch.

### Assessment of frailty

The primary outcome was frailty, and frailty was measured by frailty index (FI). Following a standard procedure (21), we employed 46 health deficits, namely, self-reported health, psychological profile, activities of daily living, instrumental activities of daily living, vision or hearing function, cognitive ability, cardiac rhythm, chronic diseases, and interviewer-rated health status, to generate the FI (Supplementary Table S1). These items were comparable to ones used in prior investigations (22–24). Each item was mapped to range from 0 (deficit absent) to 1 (deficit present) for 45 of 46 items. For the remaining item, a score of 2 was assigned if ≥2 serious illnesses were present, or being bed-ridden for the past 2 years (Supplementary Table S1). The FI was computed as the identified deficits divided by the sum of potential deficits, yielding a continuous score of 0 to 1. In case of participants with missing deficit data, we eliminated the missing deficits from both the denominator and numerator (24, 25). If over 30% of deficits were missing, we then reported the corresponding FI as missing (24). Frailty was defined as FI >0.21, and non-frailty as FI ≤ 0.21 (26, 27).

In addition to FI, the FRAIL scale (28) was further adopted to define frailty, and it included five questions assessing the presence of fatigue, resistance, ambulation, illness, and weight loss (29). As in the previous study (30), some modifications were made to the indicators of FRAIL scale in accordance with the CLHLS design. Fatigue was determined by the following question: “Do you feel the older you get, the more useless you are?”, and responses of “always” or “often” were scored as 1. Resistance was evaluated by asking “Can you continuously crouch and standup three times?”, with “unable to do so” or “a little difficult” being scored as 1. Ambulation was assessed by “Can you walk continuously for one kilometer at a time by yourself?”, and 1 point was awarded for “unable to do so” or “a little difficult”. Illness was scored 1 if subjects reported ≥5 illnesses out of 11 diseases. Weight loss was assigned 1 point for participants with BMI <18.5 kg/m^2^. The FRAIL scale items scoring criteria were detailed in Supplementary Table S2. The total score of the FRAIL scale ranged from 0 to 5, and scores ≥3 were defined as frailty, 1–2 as prefrailty, and 0 as robustness (29).

### Covariates

The following covariates were assessed: demographic characteristics (age, sex, ethnicity, marital status), socioeconomic status (educational level, residence place, primary lifetime occupation, economic independence, self-rated family economic status, and household annual income), lifestyle-related factors (smoking and drinking habit, regular physical exercise, and body mass index [BMI]).

In this study, ethnicity was classified as Han Chinese or ethnic minorities (Hui, Zhuang, Yao, Korea, Man, and others). Marital status was divided into married or non-married (divorced, widowed, and never married). Education level was dichotomized into no schooling or ≥1 year of schooling. The residence place was dichotomized as rural or urban regions. Primary lifetime occupation was based on the self-reported primary employment, and was classified into white collar (professional, governmental, commercial, industrial, and military personnel) and others occupation (self-employed, agricultural personnel, houseworkers and those never worked). Economic independence was identified if the primary financial source was from the participants' own work or pension, rather than from government subsidies, children, or other sources. Self-rated family economic status was assessed by the question “How do you rate your economic status compared with others in your local area?”, and was defined as rich if participants answered “very rich” or “rich”, medium if “so so”, and poor if “poor” or “very poor”. Household annual income (yuan) was classified into three groups: ≤ 10,000, 10,001–30,000, and >30,000. Smoking status was evaluated using the following two questions: “whether smoke at present” and “whether smoked in the past”. Those smoked regularly at the time of interview were regarded as current smoking, those with a smoking history were considered to be past smoking, and those never smoked were regarded as never smoking. A similar approach was taken to define drinking status. BMI was calculated as weight/height^2^ (kg/m^2^), and was divided into four groups: underweight <18.5, normal 18.5–23.9, overweight 24.0–27.9, and obese ≥28 kg/m^2^.

As missing values existed in most covariates except for age, sex and residence, to account for missing data (between 0.17% and 10.02%), multiple imputations (MI) were conducted by using the chained equation method with five replications. The distribution of the observed complete-case and imputed data was similar for all covariates (Supplementary Table S3).

### Statistical analysis

Continuous variables were presented as mean with standard deviation (SD), and were assessed with the Student's *t*-test; whereas categorical variables were expressed as numbers with percentages, and were evaluated *via* the χ^2^ or Fisher's exact test, as appropriate.

We computed the crude incidence rate (per 100 person-years) of frailty in relation to cooking fuel types. Then, the Cox proportional hazards models were used to explore the association between cooking fuels and incident frailty. The survival time was calculated from the baseline interview date to the date of death or the end of the study (Dec 1, 2014). The following models were sequentially generated: (1) unadjusted; (2) Model 1: adjusted for age and sex; (3) Model 2: additionally adjusted for marital status, ethnicity, education, residence, occupation, economic independence, family economic status, household income, smoking and drinking habit, regular exercise, and BMI. The results were expressed as pooled hazard ratios (HRs) with 95% confidence intervals (CIs) obtained after MI.

Stratified analyses were conducted to examine the possible modifications by age, sex, marital status, ethnicity, education level, residence, occupation, financial status, smoking and drinking habit, regular exercise, and BMI categories. The interactions between cooking fuels and stratified factors were assessed by adding the multiplicative term in the multivariate Cox model. In addition, an exploratory analysis was performed to investigate the potential impact of switching cooking fuel types on frailty risk.

Several sensitivity analyses were further conducted to assess the robustness of our results. First, a competing risk model was carried out to explore the relationship between cooking fuels and frailty, in which death without frailty was regarded as a competitive event. Second, we performed the complete-case analysis to examine the potential effect of MI. Third, we additionally corrected for geographic location (central/eastern/northeastern/northern/northwestern/ southern/southwestern), co-residence (residing with family members/living alone or in an institution), regular physical labor (yes/no), central obesity (yes [waist circumference ≥85 cm in men or ≥80 cm in women]/no), and sleep quality (good/so so/bad), as these variables may also affect frailty.

Stata 15.0 (StataCorp, College Station, TX, USA) was employed for all data analyses, and a 2-sided *P* < 0.05 was considered statistically significant.

## Results

### Baseline characteristics

Among the 9,765 individuals included in the 2011/2012 survey, we identified 8,687 clean or biomass cooking fuel users. According to the exclusion criteria, 4,643 eligible participants were ultimately entered into the FI analysis, and 5,257 participants into the FRAIL scale (Figure 1). The excluded subjects were older and had higher baseline FI than those included (all *P* < 0.05, Supplementary Table S4). The average age of 4,643 eligible participants was 80.9 ± 9.6 years, and 53.7% were male (Table 1). In comparison with clean fuel users, subjects using biomass fuels were more likely to be married, ethnic minorities, illiterate, smoker, reside in rural areas, have non-professional work, be financially dependent, have lower economic level, not exercise regularly, and have lower BMI (all *P* < 0.05).

**Table 1:** Characteristics of participants according to cooking fuel types at baseline.

**Characteristic**	**Total (*n* = 4,643)**	**Clean fuels (*n* = 2,345)**	**Biomass fuels (*n* = 2,298)**	***P***
Age, mean (SD)	80.9 (9.6)	81.0 (9.5)	80.9 (9.6)	0.70
Male, *n* (%)	2,495 (53.74)	1,229 (52.41)	1,266 (55.09)	0.07
Married, *n* (%)	2,341 (50.54)	1,139 (48.68)	1,202 (52.44)	0.01
Ethnicity, *n* (%)				<0.01
Han Chinese	3,952 (93.52)	2,132 (95.82)	1,820 (90.95)	
Ethnic minorities	274 (6.48)	93 (4.18)	181 (9.05)	
No schooling, *n* (%)	2,254 (48.64)	999 (42.69)	1,255 (54.71)	<0.01
Rural residence, *n* (%)	2,476 (53.33)	871 (37.14)	1,605 (69.84)	<0.01
Primary lifetime occupation, *n* (%)				<0.01
White collar	915 (21.90)	761 (34.51)	154 (7.81)	
Others	3,263 (78.10)	1,444 (65.49)	1,819 (92.19)	
Economic independence, *n* (%)	1,635 (35.28)	977 (41.72)	658 (28.70)	<0.01
Self-rated family economic status, *n* (%)				<0.01
Rich	897 (19.40)	613 (26.21)	284 (12.43)	
Medium	3,128 (67.65)	1,553 (66.40)	1,575 (68.93)	
Poor	599 (12.95)	173 (7.40)	426 (18.64)	
Household annual income (yuan), *n* (%)				<0.01
≤ 10,000	1,918 (45.18)	614 (28.92)	1,304 (61.45)	
10,001–30,000	1,236 (29.12)	672 (31.65)	564 (26.58)	
>30,000	1,091 (25.70)	837 (39.43)	254 (11.97)	
Smoking status, *n* (%)				<0.01
Never smoking	2,796 (60.45)	1,430 (61.14)	1,366 (59.76)	
Past smoking	735 (15.89)	401 (17.14)	334 (14.61)	
Current smoking	1,094 (23.65)	508 (21.72)	586 (25.63)	
Drinking status, *n* (%)				<0.01
Never drinking	2,931 (63.54)	1,450 (62.37)	1,481 (64.73)	
Past drinking	655 (14.20)	370 (15.91)	285 (12.46)	
Current drinking	1,027 (22.26)	505 (21.72)	522 (22.81)	
Regular exercise, *n* (%)	2,027 (44.13)	1,325 (57.01)	702 (30.94)	<0.01
BMI, mean (SD)	22.58 (28.46)	23.66 (39.38)	21.48 (7.12)	<0.01
Comorbidity, *n* (%)				
Hypertension	1,242 (27.57)	725 (31.65)	517 (23.35)	<0.01
Diabetes	180 (4.02)	128 (5.64)	52 (2.35)	<0.01
Heart disease	415 (9.25)	283 (12.43)	132 (5.97)	<0.01
Stroke or CVD	212 (4.71)	128 (5.61)	84 (3.78)	<0.01
Baseline frail index, mean (SD)	0.12 (0.05)	0.12 (0.05)	0.12 (0.05)	0.23
Death during follow-up	739 (15.92)	381 (16.25)	358 (15.58)	0.53

BMI, body mass index; CVD, cerebral vascular disease; SD, standard deviation.

### Association between biomass fuels usage and frailty

During the 11,340 person-years follow-up (median [interquartile range], 2.7 [2.0–2.8] years), 923 (19.9%) incident frailty was identified using FI (incidence rate, 8.14 [95% CI 7.65–8.66] per 100 person-years). Compared to clean fuels, cooking with biomass fuels was related to an enhanced frailty risk (incidence rates, 8.31 vs. 7.98 per 100 person-years; crude HR 1.22 [95% CI 1.08–1.39]) (Table 2). After adjustments for age and sex, biomass fuels usage was still positively associated with frailty (Model 1: HR 1.22, 95% CI 1.07–1.39). The association remained significant even after full adjustment of potential covariates (Model 2: HR 1.23, 95% CI 1.06–1.43). Similar relationships were observed between biomass fuels and frailty assessed by the FRAIL scale: HR 1.45 (95% CI 1.24–1.70) in unadjusted model, HR 1.45 (95% CI 1.24–1.71) in Model 1, and HR 1.24 (95% CI 1.04–1.50) in Model 2 (Table 2).

**Table 2:** The association between cooking fuels and frailty.

**Groups**	**Number of events/Incidence rate per 100 person-years (95% CI)**	**Unadjusted**		**Model 1**		**Model 2**	
		**HR (95% CI)**	***P***	**HR (95% CI)**	***P***	**HR (95% CI)**	***P***
**Frailty ascertained by the frailty index**							
Clean fuels	469/7.98 (7.31–8.70)	Reference		Reference		Reference	
Biomass fuels	454/8.31 (7.61–9.07)	1.22 (1.08–1.39)	<0.01	1.22 (1.07–1.39)	<0.01	1.23 (1.06–1.43)	<0.01
**Frailty ascertained by the FRAIL scale** ^a^							
Clean fuels	295/4.46 (3.99–4.98)	Reference		Reference		Reference	
Biomass fuels	324/5.57 (5.01–6.19)	1.45 (1.24–1.70)	<0.001	1.45 (1.24–1.71)	<0.001	1.24 (1.04–1.50)	0.02

HR, hazard ratio; CI, confidence interval. Model 1: adjusted for age and sex; Model 2: adjusted for model 1 + marital status, ethnicity, education, residence place, primary lifetime occupation, economic independence, self-rated family economic status, household annual income, smoking status, drinking status, regular exercise, and BMI.

^a^n = 5,257, including 2,730 clean fuel users and 2,527 biomass fuel users. During 12,421 person-years of follow-up, 619 (11.8%) incident frailty was identified (incidence rate, 4.98 [95% CI 4.61–5.38] per 100 person-years).

Sensitivity analyses further supported these results. The competing risk models, in which death without frailty was included as the competing event, coincided with our primary analysis (Supplementary Tables S5, S6). The model including complete cases only and the model additionally corrected for other confounders were also in line with our initial adjusted, imputed model (Supplementary Tables S5, S6).

### Subgroup analysis

**Figure 2:**
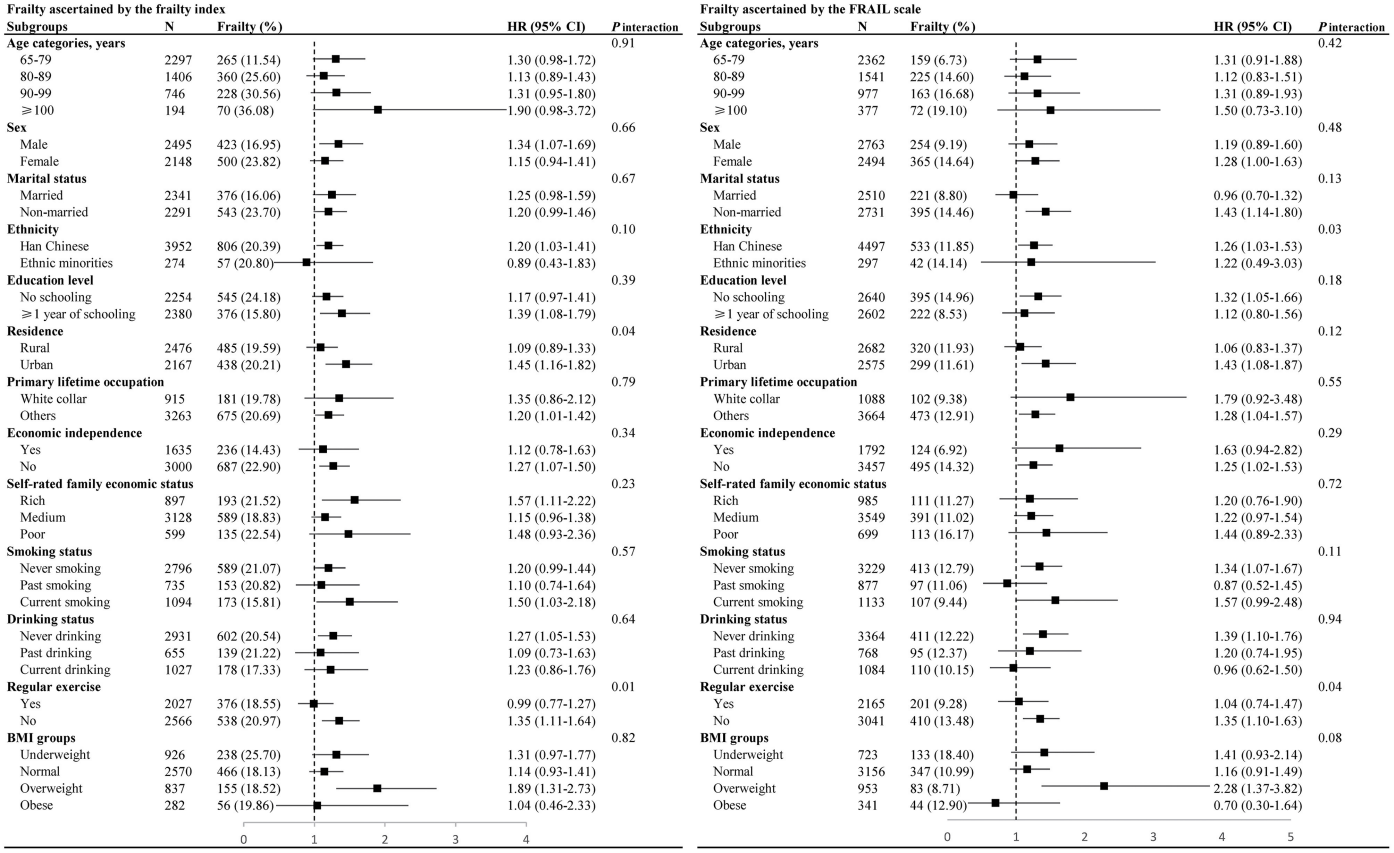
Stratified analyses to identify variables that may modify the association between biomass fuels and frailty. Hazard ratios were adjusted for age, sex, marital status, ethnicity, education, residence, primary lifetime occupation, economic independence, self-rated family economic status, household annual income, smoking status, drinking status, regular exercise, and BMI, except for the stratified variable. BMI, body mass index; HR, hazard ratio; CI, confidence interval.

The results of our stratified analyses were presented in Figure 2. We found that the place of residence modified the association between biomass cooking fuels and frailty identified by FI (*P* for interaction = 0.04, Figure 2), with the risk higher among urban residents. Further analysis revealed a significantly increased frailty risk in older individuals living in towns (HR 1.44, 95% CI 1.13–1.84) vs. cities (HR 1.60, 95% CI 0.62–4.15, Supplementary Table S7). Similarly, a marked association was present between biomass fuel use and regular exercise on frailty (*P* for interaction <0.05), and the frailty risk was markedly elevated among subjects lacking regular exercise (Figure 2).

### Switching cooking fuels and frailty

Among the 2,345 participants who used clean fuels at baseline, 1,936 (82.6%) persisted in this practice until 2014, 301 (12.8%) switched to biomass fuels, 16 (0.7%) to no cooking, 61 (2.6%) to other fuels, and 31 (1.3%) had missing cooking fuel data in 2014. Compared to persistent clean fuels, switching to biomass fuels exhibited a higher risk of frailty, as assessed by FI (incidence rates, 7.87 vs. 7.81 per 100 person-years; crude HR 1.41 [95% CI 1.06–1.87]) (Table 3). In the multivariate analysis, after adjusting for all covariates, changing from clean to biomass fuels increased frailty risk by 1.36-fold, although the association was not significant (Model 2: 95% CI 0.99–1.85). A similar relationship was observed, based on the FRAIL scale (Table 3).

**Table 3:** The association between switching cooking fuels and frailty.

**Groups**	**Number of participants**	**Number of events/incidence rate per 100 person-years (95% CI)**	**Unadjusted**		**Model 1**		**Model 2**	
			**HR (95% CI)**	***P***	**HR (95% CI)**	***P***	**HR (95% CI)**	***P***
**Switching from clean to biomass fuels**							
**Frailty index**							
Persistent clean fuels	1,936	385/7.81 (7.09–8.59)	Reference		Reference		Reference	
Clean to biomass fuels	301	54/7.87 (6.08–10.13)	1.41 (1.06–1.87)	0.02	1.20 (0.90–1.61)	0.21	1.36 (0.99–1.85)	0.06
**FRAIL scale**							
Persistent clean fuels	2246	242/4.38 (3.87–4.95)	Reference		Reference		Reference	
Clean to biomass fuels	344	33/4.34 (3.11–6.03)	1.35 (0.93–1.94)	0.11	1.10 (0.76–1.60)	0.61	1.09 (0.72–1.63)	0.69
**Switching from biomass to clean fuels**							
**Frailty index**							
Persistent biomass fuels	1497	274/7.74 (6.90–8.67)	Reference		Reference		Reference	
Biomass to clean fuels	680	140/8.54 (7.28–9.99)	0.95 (0.78–1.17)	0.65	0.92 (0.75–1.13)	0.42	0.86 (0.70–1.06)	0.16
**FRAIL scale**							
Persistent biomass fuels	1634	200/5.36 (4.68–6.13)	Reference		Reference		Reference	
Biomass to clean fuels	755	93/5.27 (4.32–6.41)	0.87 (0.68–1.11)	0.27	0.79 (0.62–1.02)	0.07	0.79 (0.62–1.02)	0.07

HR, hazard ratio; CI, confidence interval. Model 1: adjusted for age and sex; Model 2: adjusted for model 1 + marital status, ethnicity, education, residence, primary lifetime occupation, economic independence, self-rated family economic status, household annual income, smoking status, drinking status, regular exercise, and BMI.

Among the 2,298 baseline biomass fuel users, 1,497 (65.1%) persisted in using biomass fuels in 2014, 680 (29.6%) switched to clean fuels, 27 (1.2%) to no cooking, 63 (2.7%) to other fuels, and 31 (1.3%) had missing data on cook fuels. Compared to persistent biomass fuels, switching to clean fuels diminished frailty risk by 14% despite the nonsignificant association (Model 2: HR 0.86, 95% CI 0.70–1.06) (Table 3).

## Discussion

In this study, we found that cooking with biomass fuels was associated with an increased risk of frailty in older adults, as assessed both by the frailty index and the FRAIL scale. And the risk was significantly higher among town residents and participants lacking regular exercise. Switching from biomass to clean fuels might decrease the frailty risk. Our findings add to the existing knowledge of air pollution, and offer evidence on the direct association between HAP from biomass fuel combustion and frailty among older adults.

Exposure to air pollution may cause a series of unfavorable health events (31), and older people are more vulnerable to the effects of air pollution than other age groups (17). Emerging evidence has demonstrated the detrimental effect of ambient air pollution on frailty in older individuals (14–17). However, limited studies have previously explored the potential link between HAP and frailty. A recent cohort, involving 2,225 older adults (mean age 67.9 ± 6.6 years) from the CHARLS, reported that solid cooking fuels are associated with an enhanced risk of phenotypic frailty (adjusted HR 1.26, 95% CI 1.03–1.55) (13), which partly agreed with our present results. Unlike the previous study (13), we enrolled more vulnerable people with older age (mean age 80.9 ± 9.6 years), and the sample size of our cohort was approximately two times that of the previous cohort. In addition to the frail index, we further selected the FRAIL scale, instead of the physical frailty phenotype (PFP) scale, to assess frailty status. Because the FRAIL scale is well established as an excellent, time- and cost-effective screening tool for frailty, which can be rapidly administered in clinical practice without use of performance measurement (as opposed to the PFP scale) (32). Moreover, we conducted a range of stratified and sensitivity analyses to test the validity of our primary results. And an exploratory analysis was also performed to assess the potential impact of switching cooking fuels on frailty risk. Our study revealed that late-life exposure to biomass fuels is a risk factor for frailty, and lowering this exposure might reduce the frailty risk.

Counterintuitively, we demonstrated a stronger association between biomass fuels and frailty among urban residents. Further analysis revealed that the association was only significant in town residents (HR 1.44, 95% CI 1.13–1.84) but not for city dwellers (HR 1.60, 95% CI 0.62–4.15, Supplementary Table S7). One possible explanation may be related to the social structure. In China, towns are considered mixed urban-rural areas (33). Compared to cities, life in towns is relatively simple, traditional, and backward (34). Our present study mirrors this in revealing that about 45% of town residents still use biomass fuels for cooking (Supplementary Table S8). In contrast to those living in rural areas, town residents, however, often have convenient transportation as well as access to more medical services for the acquirement of health diagnoses, which may, in turn, reduce the underestimation of frailty incidence among rural residents. These reasons may help explain the significant association between biomass fuel use and frailty among town residents. Our findings support the potential benefits of accelerating access to clean energy, which is especially promising for public health in low- and middle-income countries that are experiencing an unprecedented pace of urbanization. Our present study also suggests that older adults, who do not perform regular exercise, may exhibit a higher frailty risk when expose to biomass fuels. This may be attributed to the fact that exercise can preserve or improve the function of multiple physiological systems (e.g., neuromuscular and cardiopulmonary function, cognition, and endocrine system) (1), which may partly offset the harmful effect of biomass fuels.

Although statistically non-significant, our exploratory analyses did find that switching cooking fuels from biomass to clean fuels displayed a trend toward reducing the frailty risk. The opposite effect was observed when changing from clean to biomass fuels. These results imply the potential advantages of improving household air environment in preventing or slowing frailty. Contrarily, worsening indoor air environment may exacerbate the frailty conditions among older adults. Given the potential reversibility of frailty (1), our findings may provide the public health implications for creating a healthier aging society in China.

The potential mechanisms underlying biomass fuels and frailty remain unclear, and may be related to fine particulate matter (PM). The burning of biomass fuels produces detrimental chemicals and particles causing HAP, particularly when they are combusted in inefficient cooking stoves (35). It is estimated that the incomplete combustion of solid fuels emits about 10%−38% of their fuel carbon into the air (36). These released substances may induce chronic inflammation, oxidative stress, metabolic alterations, as well as genetic and epigenetic modifications, which can destroy cellular and molecular structures (31), consequently contributing to the cumulative decline in multiple physiological systems. Previous investigations have demonstrated the positive association of HAP from biomass fuel use with cardiovascular diseases (37), respiratory disease (38), cognitive impairment (39), and psychological disorders (40). And the disruption of multi-systemic homeostasis would ultimately contribute to frailty while hampering successful aging.

It is worth noting that household activities such as heating can also cause HAP (38) and result in harmful exposure. Some recent studies have revealed that indoor solid fuels for heating were associated with a higher risk of disability (41) and mortality (42). It can be postulated that the dual exposure to HAP from cooking and heating may exhibit overlapping effects and pose a persistent danger to human health. As data on household heating fuels was lacking in the CLHLS, we were unable to explore the potential impact of heating fuels on frailty. Future studies are needed to take HAP exposure from all domestic activities into consideration. Moreover, mixed use of multiple fuels is common in China, especially in rural families (43). The majority of households prefer to use one primary fuel type while selecting a secondary type as a standby (44). Hence, the primary fuel data may not reflect the true burden of HAP. Co-exposure to chemicals from mixed fuel combustion may cause a synergetic effect (42), and thus mixed fuel use may have more detrimental health impact than a single fuel use alone. And a greater mortality risk of cancer has been observed among mixed wood and coal fuel users, compared with those only using wood (42). Given fuel stacking data was unavailable, the relationship between mixed cooking fuels and frailty was not assessed in this study. The complex interaction between different fuel types requires further research.

With the accelerated economic and societal development, the household fuel use pattern in China may have changed substantially over the past decades. Recent data from the China Family Panel Studies illustrated the considerable change, showing a significant increasing clean cooking fuel and a declining solid cooking fuel usage from 2010 to 2018 (45). This polluting-to-clean transition trend of indoor fuel pattern may bring potential health benefits. However, the data used in this study was about 10 years ago, which may not be used for realistic estimation of household fuel pattern in China today. Hence, the association between biomass cooking fuels and frailty observed in our study might be overrated. Also of note, the lockdown policies and economic downturn associated with the COVID-19 pandemic in recent years may also influence the household energy consumption. The lockdown may increase household usages of solid fuels especially in rural areas (46). Besides, during the lockdown, people were confined to their homes, further extending the exposure time to HAP. It has been reported that lockdown measures during the pandemic significantly increased indoor PM_2.5_ exposure (46). Furthermore, COVID-19 infection itself may negatively affect human health. A recent study has indicated that the pandemic decreased the physical activity of older adults and was independently related to a higher incidence of frailty (47). As the CLHLS has not yet released data on 2021 wave, we were unable to explore the potential effect of COVID-19. Our present study may underestimate the true relationship between biomass cooking fuels and frailty following the COVID-19. Further studies are warranted to elucidate the historical change in household fuel pattern and the impact of COVID-19 pandemic.

Our investigation has multiple strengths. First, to our knowledge, this is the largest prospective study examining the association between HAP from biomass fuels and frailty. The large sample size enabled us to carry out expansive subgroup and sensitivity analyses. Second, our study population was older people aged ≥65 years (50.5% of participants ≥80 years). This demographic was vulnerable to air pollution, rarely migrated, and hardly changed their socioeconomic status, thus improving the relevance and generalizability of our results. Third, we applied comprehensive and well-validated assessment tools (both the frailty index and the FRAIL scale) to measure the frailty status, which further strengthens our conclusion. Finally, MI was conducted to deal with missing data, and the complete-cases analyses further supported the reliability of our results. And we also adjusted for multiple risk factors that may affect our results, namely, socioeconomic status, behavior and lifestyle-related factors, geographic region, and other potential confounders, which may aid in the detection of true relationships.

However, some limitations must be acknowledged. First, our participants were exclusively Chinese older adults, limiting the generalizability of our results to young individuals and other populations. Second, household fuel use was self-reported, which may introduce potential recall bias. Besides, we only used primary cooking fuel types as the exposure, and lacking information on fuel stacking may cause exposure misclassification. Directly measure the concentrations of household air pollutants would be more objective. Third, other HAP sources (e.g., fuels use for heating and lighting, and second-hand smoke) and outdoor air pollution were not evaluated due to data unavailability, and therefore residual confounding cannot be excluded. Future studies are required to comprehensively consider the impact of outdoor air pollution and other indoor air pollution sources. Fourth, it was reported that the duration of solid fuels exposure may influence older people's health (48). Nevertheless, in this study, we had insufficient data to precisely assess how long the cooking fuels have been used, and similarly, we also did not know the exact timing of the cooking fuel switch during follow-up, all of which may potentially bias our results. Fifth, the effects of kitchen ventilation facilities and ventilation quality on frailty were not assessed due to lack of relevant data. Six, household fuel choice is strongly related to socioeconomic status (44), which itself can affect the development of frailty (1). Although we have adjusted for several related variables, residual confounding from unmeasured socioeconomic factors may still exist. Finally, the hazardous effect of HAP from biomass fuel usage may be chronic. The relatively short follow-up time of this study (median 2.7 years) may underestimate the association between biomass cooking fuels and frailty. Our study therefore should be considered preliminary. More evidences with longer follow-ups are warranted to examine the possible effects of HAP from biomass fuels, and confirm its association with frailty.

## Conclusions

Cooking with biomass fuels was associated with an increased frailty risk in older adults. And this risk was significantly higher among individuals living in town and those lacking regular exercise. Switching from biomass to clean fuels might reduce the frailty risk. Household fuel use pattern in China may have changed dramatically over the past decades with the rapid socioeconomic development and the efforts in policy. The pandemic of COVID-19, instead, may exacerbate household air pollution and pose challenges in access to clean energy, especially in rural areas. Though our present study may not realistically depict the household environment situation in China today, solid fuels (mainly biomass) might currently remain the major fuel for most households in rural China. Our study add evidence on the association between biomass cooking fuels and frailty among older adults. Our findings suggest that improving cooking fuels and access to clean energy might lower the risk of frailty among older people. Further researches are required to validate our results and to elucidate the potential benefits of reducing household biomass fuel use.

## Data availability statement

The raw data supporting the conclusions of this article will be made available by the authors, without undue reservation.

## Ethics statement

The studies involving human participants were reviewed and approved by Peking University, Beijing, China (IRB00001052-13074). The patients/participants provided their written informed consent to participate in this study.

## Author contributions

QSo: conceptualization, methodology, data curation, formal analysis, software, and writing—original draft. MD: visualization, methodology, data curation, software, and writing—review and editing. TL: visualization, methodology, software, and writing—review and editing. YZ: visualization and writing—review and editing. XP and RL: writing—review and editing. QSu: conceptualization, project administration, and writing—review and editing. JY: conceptualization, funding acquisition, project administration, and writing—review and editing. All authors contributed to the article and approved the submitted version.
