# Supplementary material for: Biomass fuel usage for cooking and frailty among older adults in China: a population-based cohort study
Source: Front Public Health. 2023 Apr 12;11:1122243. doi: 10.3389/fpubh.2023.1122243 (PMC10131187; doi:10.3389/fpubh.2023.1122243)
Supplement: Supplementary file 1 [file Data_Sheet_1.pdf]

Biomass fuel usage for cooking and frailty among older adults in China: a population-based cohort study

Quhong Song[1#], Miao Dai[2#], Taiping Lin[1#], Yanli Zhao[1], Xuchao Peng[1], Rui Liang[1], Qiaoli Su[3*], Jirong Yue[1*]

[1]Department of Geriatrics and National Clinical Research Center for Geriatrics, West China Hospital, Sichuan University, Chengdu, China

[2]Department of Geriatrics, Jiujiang First People's Hospital, Jiujiang, China

[3]Department of General Practice, West China Hospital, Sichuan University, Chengdu, China

[#]These authors contributed equally.

[*] **Corresponding author:** Professor Jirong Yue, Department of Geriatrics and National Clinical Research Center for Geriatrics, West China Hospital, Sichuan University, No. 37, Guo Xue Xiang, Chengdu, 610041, Sichuan, China. E-mail: yuejirong11@hotmail.com. Tel: +086 18980601143; and Professor Qiaoli Su, Department of General Practice, West China Hospital, Sichuan University, No. 37, Guo Xue Xiang, Chengdu, 610041, Sichuan, China. E-mail: 2961774468@qq.com. Tel: +086 13018290790.

Supplementary materials

Table S1 The items included in the Frailty Index

No.	Variables	Values
1	Self-reported health	Very bad= 1, Bad= 0.75, So so= 0.5, Good= 0.25, Very good= 0
2	Health worsened in the past year	Much worse= 1, A little worse= 0.75, No change= 0.5, A little better= 0.25, Much better= 0
3	Look on the bright side of things	Never= 1, Seldom= 0.75, Sometimes= 0.5, Often= 0.25, Always= 0
4	Keep my belongings neat and clean	Never= 1, Seldom= 0.75, Sometimes= 0.5, Often= 0.25, Always= 0
5	Feel fearful or anxious	Always= 1, Often=0.75, Sometimes= 0.5, Seldom= 0.25, Never= 0
6	Make own decision	Never= 1, Seldom= 0.75, Sometimes= 0.5, Often= 0.25, Always= 0
7	Feel useless with age	Always= 1, Often=0.75, Sometimes= 0.5, Seldom= 0.25, Never= 0
8	Do house work at present	Never= 1; Not monthly, but sometimes= 0.5; Not weekly, but at least once a month= 0.5; Not daily, but at least once a week=0.5; Almost every day=0
9	ADLs: Needs assistance bathing	More than one part assistance= 1, One part assistance= 0.5, Without assistance= 0
10	ADLs: Needs assistance dressing	Assistance in getting clothes and getting dressed= 1, Need assistance for trying shoes= 0.5, Without assistance= 0
11	ADLs: Needs assistance toileting	Don't use toilet= 1, Assistance in cleaning or arranging clothes= 0.5, Without assistance= 0
12	ADLs: Needs assistance in indoor transferring	Bedridden= 1, With assistance= 0.5, Without assistance= 0
13	ADLs: Incontinence	Incontinent= 1, Occasional accidents= 0.5, Without assistance= 0
14	ADLs: Needs assistance eating	Need feeding= 1, With some help= 0.5, Without assistance= 0
15	IADLs: able to visit neighbors by oneself	Unable to do so= 1, A little difficult= 0.5, Yes= 0
16	IADLs: able to shop by oneself if necessary	Unable to do so= 1, A little difficult= 0.5, Yes= 0
17	IADLs: able to make food by oneself if necessary	Unable to do so= 1, A little difficult= 0.5, Yes= 0
18	IADLs: able to wash clothing by oneself	Unable to do so= 1, A little difficult= 0.5, Yes= 0
19	IADLs: able to walk continuously for 1 kilometer	Unable to do so= 1, A little difficult= 0.5, Yes= 0
20	IADLs: able to lift a weight of 5 kg (such as a heavy bag of groceries)	Unable to do so= 1, A little difficult= 0.5, Yes= 0
21	IADLs: able to continuously crouch and stand up three times	Unable to do so= 1, A little difficult= 0.5, Yes= 0
22	IADLs: able to use public transportation	Unable to do so= 1, A little difficult= 0.5, Yes= 0

23	Vision loss	Can't see or blind= 1, Can see only= 0.5, Can see and distinguish= 0
24	Hearing loss	Yes= 1, No= 0
25	Able to use chopsticks to eat	No= 1, Yes= 0
26	Able to put hand behind neck	Neither hand= 1, Right or left hand only= 0.5, Both hands= 0
27	Able to put hand behind lower back	Neither hand= 1, Right or left hand only= 0.5, Both hands= 0
28	Able to raise arm upright	Neither hand= 1, Right or left hand only= 0.5, Both hands= 0
29	Able to stand up from sitting in a chair	No= 1; Yes, using hands= 0.5; Yes, without using hands= 0
30	Able to pick up a book from the floor	No= 1; Yes, sitting= 0.5; Yes, standing= 0
31	Heart rhythm	Irregular= 1, Regular= 0
32	Steps used to turn around 360 with help?	Cannot turn around= 1, >4= 0.5, ≤4= 0
33	Number of serious illnesses in the past 2 years	Two or more illnesses or bedridden= 2, one illness= 1, No= 0
34	Suffering from hypertension	Yes= 1, No= 0
35	Suffering from diabetes	Yes= 1, No= 0
36	Suffering from heart disease	Yes= 1, No= 0
37	Suffering from stroke/cerebrovascular disease	Yes= 1, No= 0
38	Suffering from bronchitis, emphysema, asthma, or pneumonia	Yes= 1, No= 0
39	Suffering from tuberculosis	Yes= 1, No= 0
40	Suffering from cancer	Yes= 1, No= 0
41	Suffering from gastric or duodenal ulcers	Yes= 1, No= 0
42	Suffering from Parkinson's disease	Yes= 1, No= 0
43	Suffering from bedsore	Yes= 1, No= 0
44	Suffering from arthritis	Yes= 1, No= 0
45	Dementia	Yes= 1, No= 0
46	Interviewer-rated health	Very unhealthy= 1, Moderately unhealthy= 0.67, Relatively healthy= 0.33, Surprisingly healthy= 0

ADLs, activities of daily living; IADLs, instrumental activities of daily living.

Table S2 The FRAIL scale scoring criteria

No.	Items	Questions	Values
1	Fatigue	Do you feel the older you get, the more useless you are?	Always or often= 1; Never, seldom, or sometimes= 0
2	Resistance	Can you continuously crouch and stand up three times?	Unable to do so or a little difficult= 1, Yes= 0
3	Ambulation	Can you walk continuously for 1 kilometer at a time by yourself?	Unable to do so or a little difficult= 1, Yes= 0
4	Illness	Self-reporting 5 or more illnesses out of total 11 illnesses [a]	≥5 = 1, <5 = 0
5	Weight loss	BMI (weight (in kilograms)/height (in meters) ^{2})	<18.5 =1, ≥18.5 =0

BMI, body mass index;

[a] The total 11 diseases included hypertension, diabetes, cancer, chronic lung disease, heart attack, congestive heart failure, angina, asthma, arthritis, stroke, and kidney disease.

Table S3 Distributions of the observed complete case data and the imputed data

Characteristics	Number (%) with missing data	Complete case	Multiple imputation 1	Multiple imputation 2	Multiple imputation 3	Multiple imputation 4	Multiple imputation 5
Ethnicity (%)	417 (8.98)						
Han Chinese		93.73	93.39	93.43	93.32	93.30	93.45
Ethnic minorities		6.27	6.61	6.57	6.68	6.70	6.55
Marital status (%)	11 (0.24)						
Married		50.21	50.57	50.59	50.57	50.55	50.57
Non-married		49.79	49.43	49.41	49.43	49.45	49.43
Education level (%)	9 (0.19)						
No schooling		48.42	48.63	48.63	48.59	48.65	48.70
≥1 year of schooling		51.58	51.37	51.37	51.41	51.35	51.30
Primary lifetime occupation (%)	465 (10.02)						
White collar		22.29	20.91	20.96	20.76	20.78	20.91
Others		77.71	79.09	79.04	79.24	79.22	79.09
Economic independence (%)	8 (0.17)						
Yes		35.95	35.26	35.28	35.26	35.26	35.26
No		64.05	64.74	64.72	64.74	64.74	64.74
Self-rated family economic status (%)	19 (0.41)						
Rich		19.53	19.38	19.38	19.38	19.41	19.43
Medium		67.06	67.65	67.65	67.65	67.72	67.59
Poor		13.41	12.97	12.97	12.97	12.92	12.99
Household annual income (yuan), n (%)	398 (8.57)						
≤10000		45.18	45.72	45.23	44.78	45.81	45.23
10001-30000		29.12	28.97	29.10	29.40	28.93	29.08
>30000		25.70	25.31	25.67	25.82	25.26	25.69
Smoking status (%)	18 (0.39)						
Never smoking		59.04	60.54	60.52	60.48	60.50	60.48
Past smoking		16.82	15.85	15.85	15.85	15.92	15.87
Current smoking		24.14	23.61	23.63	23.67	23.58	23.65
Drinking status (%)	30 (0.65)						

Never drinking		61.81	63.58	63.58	63.45	63.56	63.60
Past drinking		15.35	14.17	14.17	14.21	14.24	14.17
Current drinking		22.84	22.25	22.25	22.33	22.21	22.23
Regular exercise (%)	50 (1.08)						
Yes		47.40	44.02	44.00	44.15	44.13	44.11
No		52.60	55.98	56.00	55.85	55.87	55.89
BMI, mean (SD)	28 (0.60)	22.57 (0.47)	22.65 (0.42)	22.62 (0.42)	22.62 (0.42)	22.67 (0.42)	22.62 (0.42)

BMI, body mass index; SD, standard deviation.

Table S4 Baseline characteristics between the excluded patients and those included

Characteristics	Total (n=9765)	Excluded (n=5122)	Included (n=4643)	*P*
Age, mean (SD)	85.8 (11.4)	90.2 (11.1)	80.9 (9.6)	<0.01
Male, n (%)	4398 (45.04)	1903 (37.15)	2495 (53.74)	<0.01
Married, n (%)	3698 (38.25)	1357 (26.95)	2341 (50.54)	<0.01
Ethnicity, n (%)				0.02
Han Chinese	8423 (94.13)	4471 (94.68)	3952 (93.52)	
Ethnic minorities	525 (5.87)	251 (5.32)	274 (6.48)	
No schooling, n (%)	5652 (58.12)	3398 (66.76)	2254 (48.64)	<0.01
Rural residence, n (%)	5145 (52.69)	2669 (52.11)	2476 (53.33)	0.23
Primary lifetime occupation, n (%)				<0.01
White collar	1816 (20.56)	901 (19.35)	915 (21.90)	
Others	7018 (79.44)	3755 (80.65)	3263 (78.10)	
Economic independence, n (%)	2688 (27.77)	1053 (20.88)	1635 (35.28)	<0.01
Self-rated family economic status, n (%)				<0.01
Rich	1647 (17.17)	750 (15.09)	897 (19.40)	
Medium	6456 (67.29)	3328 (66.96)	3128 (67.65)	
Poor	1491 (15.54)	892 (17.95)	599 (12.95)	
Household annual income (yuan), n (%)				<0.01
≤10000	3773 (42.74)	1855 (40.48)	1918 (45.18)	
10001-30000	2738 (31.01)	1502 (32.77)	1236 (29.12)	
>30000	2317 (26.25)	1226 (26.75)	1091 (25.70)	
Smoking status, n (%)				<0.01
Never smoking	6369 (65.88)	3573 (70.85)	2796 (60.45)	
Past smoking	1566 (16.20)	831 (16.48)	735 (15.89)	
Current smoking	1733 (17.93)	639 (12.67)	1094 (23.65)	
Drinking status, n (%)				<0.01
Never drinking	6575 (68.30)	3644 (72.68)	2931 (63.54)	
Past drinking	1412 (14.67)	757 (15.10)	655 (14.20)	
Current drinking	1640 (17.04)	613 (12.23)	1027 (22.26)	
Regular exercise, n (%)	3181 (33.17)	1154 (23.09)	2027 (44.13)	<0.01

BMI, mean (SD)	21.98 (21.47)	21.41 (10.97)	22.58 (28.46)	<0.01
Comorbidity, n (%)				
Hypertension	2752 (28.18)	1510 (29.48)	1242 (26.75)	<0.01
Diabetes	400 (4.10)	220 (4.30)	180 (3.88)	<0.01
Heart disease	1175 (12.03)	760 (14.84)	415 (8.94)	<0.01
Stroke or CVD	802 (8.21)	590 (11.52)	212 (4.57)	<0.01
Baseline frail index, mean (SD)	0.22 (0.15)	0.32 (0.15)	0.12 (0.05)	<0.01

BMI, body mass index; CVD, cerebral vascular disease; SD, standard deviation.

Table S5 Sensitive analysis of the association between cooking fuels and frailty ascertained by the frailty index

Groups	Unadjusted model		Model 1		Model 2	
	HR (95% CI)	*P*	HR (95% CI)	*P*	HR (95% CI)	*P*
**Competing risk model**						
Clean fuels	Reference		Reference		Reference	
Biomass fuels	1.18 (1.04-1.34)	<0.01	1.17 (1.03-1.33)	0.02	1.16 (1.00-1.34)	0.04
**Complete cases** [a]						
Clean fuels	Reference		Reference		Reference	
Biomass fuels	1.22 (1.08-1.39)	<0.01	1.22 (1.07-1.39)	<0.01	1.21 (1.02-1.44)	0.03
**Additional adjustment** [b]						
Clean fuels	Reference		Reference		Reference	
Biomass fuels	1.22 (1.08-1.39)	<0.01	1.22 (1.07-1.39)	<0.01	1.23 (1.06-1.43)	<0.01

HR, hazard ratio; CI, confidence interval. Model 1: adjusted for age and sex; Model 2: adjusted for model 1 + marital status, ethnicity, education, residence, primary lifetime occupation, economic independence, self-rated family economic status, household annual income, smoking status, drinking status, regular exercise, and BMI;

[a] n=3658, including 1920 clean fuel users and 1738 biomass fuel users at baseline. 733 (20.0%) incident frailty was identified during follow-up;

[b] Model 2: additionally controlled for geographic region, co-residence, regular physical labor, central obesity, and sleep quality.

Table S6 Sensitive analysis of the association between cooking fuels and frailty ascertained by the FRAIL scale

Groups	Unadjusted model		Model 1		Model 2	
	HR (95% CI)	*P*	HR (95% CI)	*P*	HR (95% CI)	*P*
**Competing risk model**						
Clean fuels	Reference		Reference		Reference	
Biomass fuels	1.41 (1.20-1.64)	<0.001	1.37 (1.17-1.60)	<0.001	1.17 (1.00-1.41)	0.05
**Complete cases** [a]						
Clean fuels	Reference		Reference		Reference	
Biomass fuels	1.45 (1.24-1.70)	<0.001	1.45 (1.24-1.70)	<0.001	1.32 (1.07-1.63)	0.008
**Additional adjustment** [b]						
Clean fuels	Reference		Reference		Reference	
Biomass fuels	1.45 (1.24-1.70)	<0.001	1.45 (1.24-1.70)	<0.001	1.27 (1.06-1.54)	0.009

HR, hazard ratio; CI, confidence interval. Model 1: adjusted for age and sex; Model 2: adjusted for model 1 + marital status, ethnicity, education, residence, primary lifetime occupation, economic independence, self-rated family economic status, household annual income, smoking status, drinking status, regular exercise, and BMI;

[a] n= 4137, including 2232 clean fuel users and 1905 biomass fuel users at baseline. 488 (11.8%) incident frailty was identified during follow-up;

[b] Model 2: additionally controlled for geographic region, co-residence, regular physical labor, central obesity, and sleep quality.

Table S7 The association between biomass cooking fuels and frailty by the frail index, stratified by residence place

Groups	Number of participants	Number of events/Incidence rate per 100 person-years (95% CI)	Unadjusted		Model 1		Model 2	
			HR (95% CI)	*P*	HR (95% CI)	*P*	HR (95% CI)	*P*
City	692	127/7.13 (6.02-8.42)	1.75 (0.72-4.30)	0.22	1.54 (0.63-3.79)	0.34	1.60 (0.62-4.15)	0.34
Town	1475	311/8.39 (7.54-9.33)	1.29 (1.03-1.61)	0.03	1.42 (1.14-1.78)	<0.01	1.44 (1.13-1.84)	<0.01
Rural	2476	485/8.28 (7.60-9.01)	1.03 (0.86-1.24)	0.76	1.02 (0.85-1.23)	0.83	1.09 (0.89-1.33)	0.40

HR, hazard ratio; CI, confidence interval. Model 1: adjusted for age and sex; Model 2: adjusted for model 1 + marital status, ethnicity, education, primary lifetime occupation, economic independence, self-rated family economic status, household annual income, smoking status, drinking status, regular exercise, and BMI.

Table S8 Characteristics of participants according to residence place

Characteristics	City (n=692)	Town (n=1475)	Rural (n=2476)	*P*
Age, years, mean (SD)	80.09 (9.43)	81.06 (9.15)	81.04 (9.87)	0.05
Male, n (%)	362 (52.31)	808 (54.78)	1325 (53.51)	0.53
Married, n (%)	364 (52.68)	733 (49.83)	1244 (50.36)	0.45
Ethnicity, n (%)				<0.01
Han Chinese	652 (97.46)	1312 (93.85)	1988 (92.08)	
Ethnic minorities	17 (2.54)	86 (6.15)	171 (7.92)	
No schooling, n (%)	204 (29.52)	726 (49.29)	1324 (53.60)	<0.01
Primary lifetime occupation, n (%)				<0.01
White collar	455 (68.32)	273 (19.63)	187 (8.82)	
Others	211 (31.68)	1118 (80.37)	1934 (91.18)	
Economic independence, n (%)	485 (70.09)	467 (31.70)	683 (27.65)	<0.01
Self-rated family economic status, n (%)				<0.01
Rich	171 (24.78)	307 (20.84)	419 (17.03)	
Medium	463 (67.10)	968 (65.72)	1697 (68.96)	
Poor	56 (8.12)	198 (13.44)	345 (14.02)	
Household annual income (yuan), n (%)				<0.01
≤10000	83 (13.05)	604 (44.91)	1231 (54.37)	
10001-30000	211 (33.18)	384 (28.55)	641 (28.31)	
>30000	342 (53.77)	357 (26.54)	392 (17.31)	
Smoking status, n (%)				<0.01
Never smoking	419 (60.72)	884 (60.14)	1493 (60.57)	
Past smoking	149 (21.59)	240 (16.33)	346 (14.04)	
Current smoking	122 (17.68)	346 (23.54)	626 (25.40)	
Drinking status, n (%)				<0.01
Never drinking	447 (65.35)	901 (61.46)	1583 (64.27)	
Past drinking	121 (17.69)	239 (16.30)	295 (11.98)	
Current drinking	116 (16.96)	326 (22.24)	585 (23.75)	
Regular exercise, n (%)	511 (74.49)	712 (48.90)	804 (32.80)	<0.01
BMI, kg/m^2, mean (SD)	25.95 (69.36)	22.48 (13.27)	21.69 (8.01)	<0.01

Comorbidity, n (%)				
Hypertension	232 (33.53)	394 (26.71)	616 (24.88)	<0.01
Diabetes	60 (8.67)	51 (3.46)	69 (2.79)	<0.01
Heart disease	131 (18.93)	117 (7.93)	167 (6.74)	<0.01
Stroke or CVD	44 (6.36)	59 (4.00)	109 (4.40)	<0.01
Baseline biomass fuel user, n (%)	30 (4.34)	663 (44.95)	1605 (64.82)	<0.01
Switching from clean to biomass fuels during follow-up, n (%)	8 (1.16)	104 (7.05)	189 (7.63)	<0.01
Baseline frail index, mean (SD)	0.11 (0.05)	0.12 (0.05)	0.12 (0.05)	<0.01
Frailty during follow-up, n (%)	127 (18.35)	311 (21.08)	485 (19.59)	0.29

BMI, body mass index; CVD, cerebral vascular disease; SD, standard deviation.
